# Measuring ocular torsion and its variations using different nonmydriatic fundus photographic methods

**DOI:** 10.1371/journal.pone.0244230

**Published:** 2020-12-22

**Authors:** Hyunkyoo Kang, Sang Jae Lee, Hyun Jin Shin, Andrew G. Lee

**Affiliations:** 1 Department of Mechatronics and Electronic Engineering, Konkuk University Glocal Campus, Chungcheongbuk-do, Republic of Korea; 2 School of Medicine, Konkuk University, Seoul, Republic of Korea; 3 Department of Ophthalmology, Research Institute of Medical Science, Konkuk University Medical Center, Konkuk University School of Medicine, Seoul, Republic of Korea; 4 Department of Ophthalmology, Blanton Eye Institute, Houston Methodist Hospital, Houston, Texas, United States of America; 5 Department of Ophthalmology, Neurology, Neurosurgery, Weill Cornell Medicine, New York, NY, United States of America; 6 Department of Ophthalmology, University of Texas Medical Branch, Galveston, Texas, United States of America; 7 Department of Ophthalmology, UT MD Anderson Cancer Center, Houston, Texas, United States of America; 8 Department of Ophthalmology, Texas A and M College of Medicine, College Station, Texas, United States of America; 9 Department of Ophthalmology, University of Iowa Hospitals and Clinics, Iowa City, Iowa; 10 Department of Ophthalmology, Baylor College of Medicine and the Center for Space Medicine, Houston, TX, United States of America; 11 Department of Ophthalmology, University of Buffalo, Buffalo, New York, United States of America; Save Sight Institute, AUSTRALIA

## Abstract

**Purpose:**

To compare the variations in ocular torsion measurements made using different fundus photographic methods.

**Methods:**

We enrolled subjects with three conditions: (1) patients with intermittent exotropia (IXT) (*n =* 44), (2) patients with unilateral superior oblique palsy (SOP) (*n =* 10), and (3) normal subjects as controls (*n* = 85). Ocular torsion was measured by disc-center–fovea angle (DFA) using three different imaging modalities: (1) conventional fundus photography (CFP) with a 45° field of view (FV), (2) wide-field fundus photography (WFP) with a 200° FV, and (3) optical coherence tomography (OCT) with a 55° FV.

**Results:**

In the IXT group, the DFAs in the right and left eyes were 5.70±3.35° and 6.37±3.36°, respectively, for CFP, 8.39±5.24° and 8.61±3.67° for WFP, and 5.73±3.61° for 6.16±3.50° for OCT. In the SOP group, the DFAs in paretic and nonparetic eyes were 12.19±1.69° and 6.71±1.09°, respectively, for CFP, 14.29±2.36° and 8.23±3.31° for WFP, and 12.12±1.73° and 6.91±1.12° for OCT. In the control group, the DFAs in the right and left eyes were 5.39±2.65° and 5.71±3.16°, respectively, for CFP, 8.77±5.56° and 8.90±6.24° for WFP, and 5.27±2.67° and 5.72±3.20° for OCT. There was no difference between the results from CFP and OCT among the three groups. However, the torsional angle was larger when measured using WFP than the other two photographic methods (CFP and OCT) in all three groups (all *p*<0.05).

**Conclusion:**

The ocular torsion measurement varies with the fundus photographic method used to measure it. Clinicians should be careful to avoid overestimating ocular extorsion when it is evaluated using WFP.

## Introduction

Abnormal ocular torsion has been observed in a number of strabismus conditions including primary or secondary inferior oblique muscle overaction, superior oblique muscle palsy (SOP), intermittent exotropia (IXT) and skew deviation [1, 2]. Thus, assessing ocular torsion is of critical importance in various strabismic and neuro-ophthalmic disorders. Documenting the torsion not only influences the differential diagnosis and management of these patients but it can change any potential strabismus surgical plan. As an example, detecting bilateral objective cyclotropia and/or subjective cyclotropia greater than 10° may help in differentiating bilateral from unilateral SOP [3]. Also, incyclotorsion of the affected (hypertropic) eye or bilateral conjugate torsion (typically towards the hypotropic eye) helps when differentiating skew deviation from SOP [4]. The double Maddox-rod test and Lancaster red–green test are the most widely used tests worldwide to assess ocular torsion. However, the quantified results of ocular torsion are only provided in rough values.

Objective measurements of ocular torsion are most commonly performed using fundus photography [5] and more recently using infrared imaging by optical coherence tomography (OCT) [6–8]. Lee and Kim reported that when comparing the conventional method of measuring ocular torsion with fundus photographs to OCT, the OCT may be useful to automatically calculate the cyclotorsion [9]. Some clinics also use wide-field fundus photography (WFP) to assess fundus torsion. WFP has the advantage of being able to detect peripheral retinal pathology as well as the optic nerve and macula at the same time. However, discrepancies between measurement methods for ocular torsion have been reported. For example, Kothari et al. reported that the torsional angle tended to be smaller when measured using a slit-lamp biomicroscopic method than a fundus photographic method [10].

In our clinical observations we have also found discordance of the cyclotorsional angle in a particular group of patients undergoing nonmydriatic WFP. Although wide-field imaging modalities have recently increased in popularity in clinical applications, to our knowledge, whether the ocular torsion data are equivalent and interchangeable between WFP and conventional photography has not been studied previously.

Therefore, the aim of the study was to determine the cyclotorsional angle measured between three nonmydriatic fundus photographic methods: (1) conventional fundus photography (CFP) with a 45° field of view, (2) WFP with a 200° field of view, and (3) OCT with a 55° field of view in patients with normal and pathological conditions.

## Materials and methods

This retrospective case–control clinical study was conducted at the Department of Ophthalmology of Konkuk University Medical Center in Seoul, South Korea between December 2017 and February 2020. The study was approved by the Institutional Review Board (IRB) and Ethics Committee at Konkuk University Medical Center (registration number: KUH 2020-07-016), and it was conducted according to the principles expressed in the Declaration of Helsinki. Patient records were anonymized and de-identified prior to performing the analyses.

### Subjects enrollment

We obtained fundus photographs from subjects with three conditions: (1) IXT patients without vertical strabismus or oblique muscle dysfunction (IXT group, *n =* 44), (2) unilateral superior oblique palsy (SOP) patients in whom the horizontal deviation did not exceed 10 prism diopters (PD) (SOP group, *n* = 10), and (3) normal subjects attending the clinic either for screening or for a routine ophthalmological checkup (control group, *n* = 85). The control group included subjects who had no prior strabismus diagnosis or complaint and were within 2 PD of being orthophoric. In the present study, patients with IXT were included as representative of horizontal strabismus.

All of the included patients underwent a comprehensive ophthalmic evaluation by a single experienced ophthalmologist (H.J.S.), which included an indirect fundus examination. The following exclusion criteria were applied to all three groups: (1) inadequate image quality/poor photograph clarity for judging the position of the center of the fovea, (2) refractive error of >6.00 or <–6.00, (3) any optic disc pathological feature such as optic disc coloboma or hypoplasia, (4) any macula diseases affecting the fovea, such as macular edema or macular dragging/scarring, (5) amblyopia or anisometropia (difference in the spherical equivalent refraction of >2), (6) an A or V pattern of IXT, (7) previous ocular surgery such as blow-out fracture, retinal detachment, or strabismus surgery, or (8) limitation of eye movement in duction/version testing.

### Fundus torsion measurements

We objectively measured the ocular torsion in both eyes using three different nonmydriatic fundus photographic methods: (1) CFP with a 45° field of view (TRC-NW8F, Topcon, Tokyo, Japan), (2) WFC with a 200° field of view (Optos, Dunfermline, Fife, UK) and (3) and OCT with a 55° field of view (Heidelberg Spectralis, Heidelberg Engineering, Heidelberg, Germany). Each eye was investigated separately in the primary position. For the measurements requiring internal fixation (FC and OCT), the patient was asked to look at an internal fixation target, whereas for WFC the patient was asked to keep looking toward the center of a test field. The three tests were all performed on the same day, and patients were asked to stay in the same position without tilting of the head using a chin and forehead rest during every test. Both eyes of the patient remained open during the measurements [3]. Data obtained from uncooperative subjects were excluded from the analysis.

**Fig 1:**
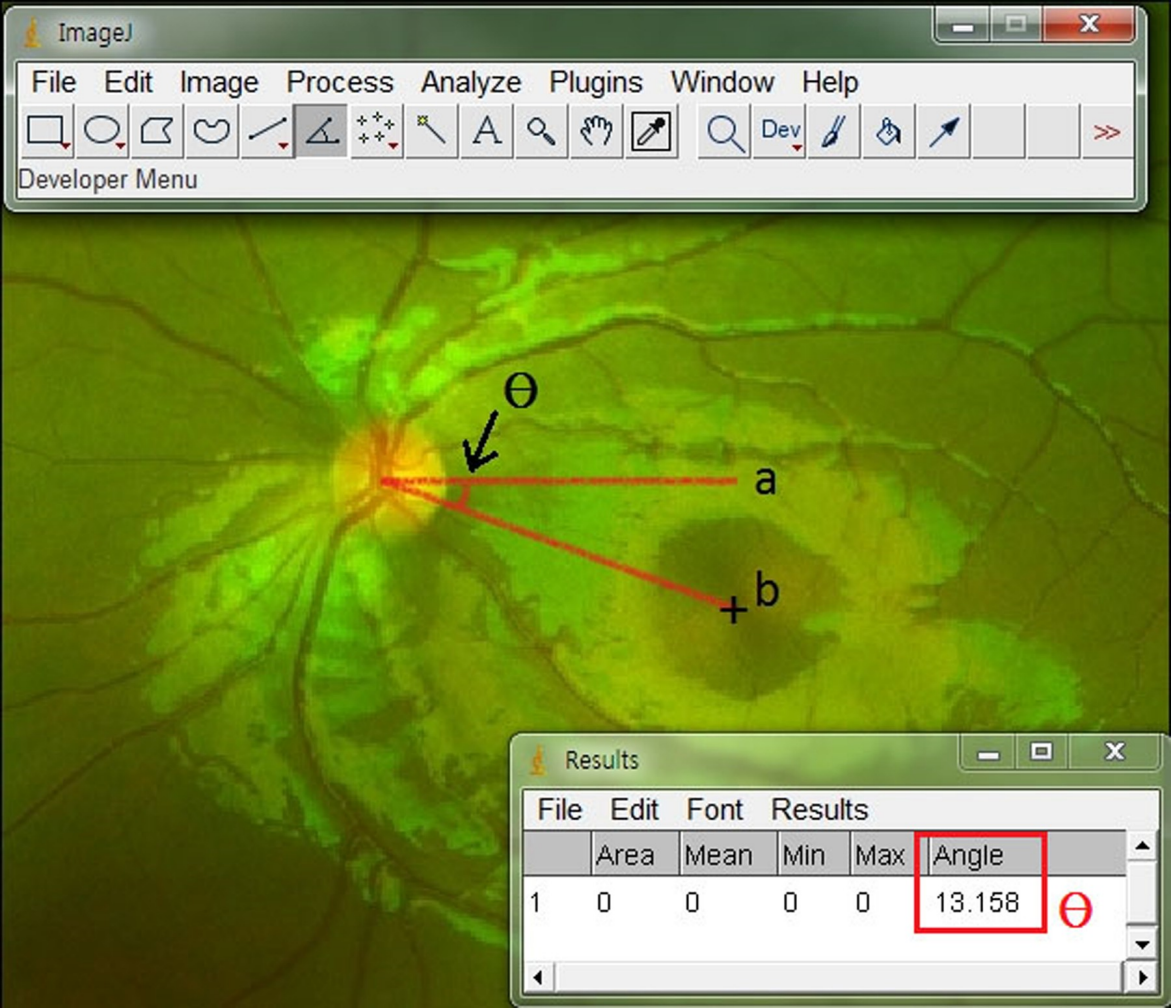
The measurement of the Disc-center–Fovea Angle (DFA) using the Image J program. ‘a’ is a horizontal line drawn from the optic disc center. ‘b’ is a line drawn from the optic disc center to the fovea. ‘*θ*’ is the angle between lines a and b.

The disc-center–fovea angle (DFA) was calculated from a single well-focused photograph. We examined the torsional angle (*θ*) between a horizontal line drawn through the optic disc center and a line connecting the optic disc center to the fovea using standard image analysis software (version 1.42q, Image J; developed by Wayne Rasbands, National Institutes of Health, Bethesda, MD, USA) (Fig 1). The center of the optic disc was defined according to previous research [11]. First, a rectangle was fitted to the height and width of the optic disc manually. Two diagonal lines were drawn, and their crossing was considered as the center. This method was used to record the amount of ocular torsion. Each image was analyzed by two experienced examiners (S.J.L. and H.J.S.) who were blinded to the patient information. They performed two measurements for each data item, with the mean value used in the subsequent analyses.

### Statistical analysis

All calculations and statistical analyses were performed using SPSS for Windows (version 19.0, SPSS, Chicago, IL, USA). Best-corrected visual acuity (BCVA) was expressed as logarithm of minimum angle of resolution (logMAR) fashion. The Shapiro-Wilk test was used to determine whether the data followed a parametric (Gaussian) or nonparametric (non-Gaussian) distribution. Statistically significant differences between photographic methods were identified using the paired *t*-test for parametric values and the Wilcoxon signed-rank test for nonparametric values. Bland-Altman plots were constructed to evaluate the agreement between the methods [12]. Comparisons of the three different photographic methods in the same patient were performed using the Kruskal-Wallis test or ANOVA with post-hoc analysis. Data are presented as mean±standard deviation values. The criterion for statistical significance was *p* < 0.05.

## Results

This study included 139 patients (278 eyes), comprising 72 males and 67 females aged 17.8±20.6 years, with an age range of 3–84 years. Each group had a median (range) BCVA (logMAR) of 0.0 (0.0 –+0.1) in right eye and left eye. The intergrader reliability for the two graders was confirmed by the к values ranging from 0.86 to 0.92 across the different variables. All measurement values (*θ*) in the three groups are listed in Table 1.

**Table 1:** Measurements of the Disc-center–Fovea Angle (DFA) in the intermittent exotropia (IXT), unilateral Superior Oblique Palsy (SOP), and control groups using different nonmydriatic photographic methods: Conventional Fundus Photography (CFP), Wide-field Fundus Photography (WFP), and Optical Coherence Tomography (OCT). Data are mean±standard-deviation values in degrees.

	IXT (*n* = 44)		SOP (*n* = 10)		Control (*n* = 85)	
	Right	Left	Paretic	Nonparetic	Right	Left
**CFP**	5.70±3.35	6.37±3.36	12.19±1.69	6.71±1.09	5.39±2.65	5.71±3.16
**WFP**	8.39±5.24	8.61±3.67	14.29±2.36	8.23±3.31	8.77±5.56	8.90±6.24
**OCT**	5.73±3.61	6.16±3.50	12.12±1.73	6.91±1.12	5.27±2.67	5.72±3.20
*p*	0.003 ^*a*^	0.002 ^*a*^	0.031 ^*a*^	0.443 ^*b*^	0.001 ^*a*^	0.001 ^*a*^

*p* value between three nonmydriatic fundus photographic methods in the same eye.

^*a*^ *p* value in ANOVA,

^*b*^ *p* value in the Kruskal-Wallis test.

The subjects in the IXT group (*n =* 44) were aged 20.1±23.1 years (range 3–82 years), and 26 (59.1%) of them were males. The distant and near horizontal deviations were 23.5±4.3 and 26.3±5.1 PD, respectively. The DFAs in the right and left eyes were 5.70±3.35° and 6.37±3.36°, respectively, for CFP, 8.39±5.24° and 8.61±3.67° for WFP, and 5.73±3.61° and 6.16±3.50° for OCT. Comparing the mean DFA between CFP and OCT did not reveal any significant differences in the right (*p* = 0.831) or left (*p* = 0.445) eyes. However, the mean DFA was significantly larger for WFP than for CFP and OCT in both eyes (all *p*<0.001).

The subjects in the SOP group (*n =* 10) were aged 42.8±21.4 years (range 3–70 years), and four (40.0%) of them were male. The distant vertical deviation was 12.2±4.1 PD. Paradoxical ocular excyclotorsion in nonparetic eyes of unilateral SOP was seen in one patient. The objective ocular torsion was paradoxical on the three imaging methods. In such a case we consider a torsional angle of the nonparetic eye as a excyclotorsion. The DFAs in paretic and nonparetic eyes were 12.19±1.69° and 6.71±1.09°, respectively, for CFP, 14.29±2.36° and 8.23±3.31° for WFP, and 12.12±1.73° and 6.91±1.12° for OCT. Comparing the mean DFA between CFP and OCT did not reveal any significant differences in paretic (*p* = 0.799) or nonparetic (*p* = 0.275) eyes. However, the mean DFA was significantly larger for WFP than for CFP and OCT in paretic eyes (*p* = 0.028 and 0.001, respectively), while there was no significant difference in nonparetic eyes (*p* = 0.160 and 0.275, respectively).

In subjects in the control group (*n* = 85) were aged 14.0±16.7 years (range 3–84 years), and 42 (49.4%) of them were male. The DFAs in the right and left eyes were 5.39±2.65° and 5.71±3.16°, respectively, for CFP, 8.77±5.56° and 8.90±6.24° for WFP, and 5.27±2.67° and 5.72±3.20° for OCT. Comparing the mean DFA between CFP and OCT did not reveal any significant differences in the right (*p* = 0.243) or left (*p* = 0.899) eyes. However, the mean DFA was significantly larger for WFP than for CFP and OCT in both eyes (all *p*<0.001). In addition, control group was divided the into three age categories. Group I (<10) n = 56; Group II (10~20) n = 19; and Group III (>20) n = 10. There was no significant age-related differences in DFA among the three group (*p* = 0.531).

**Fig 2:**
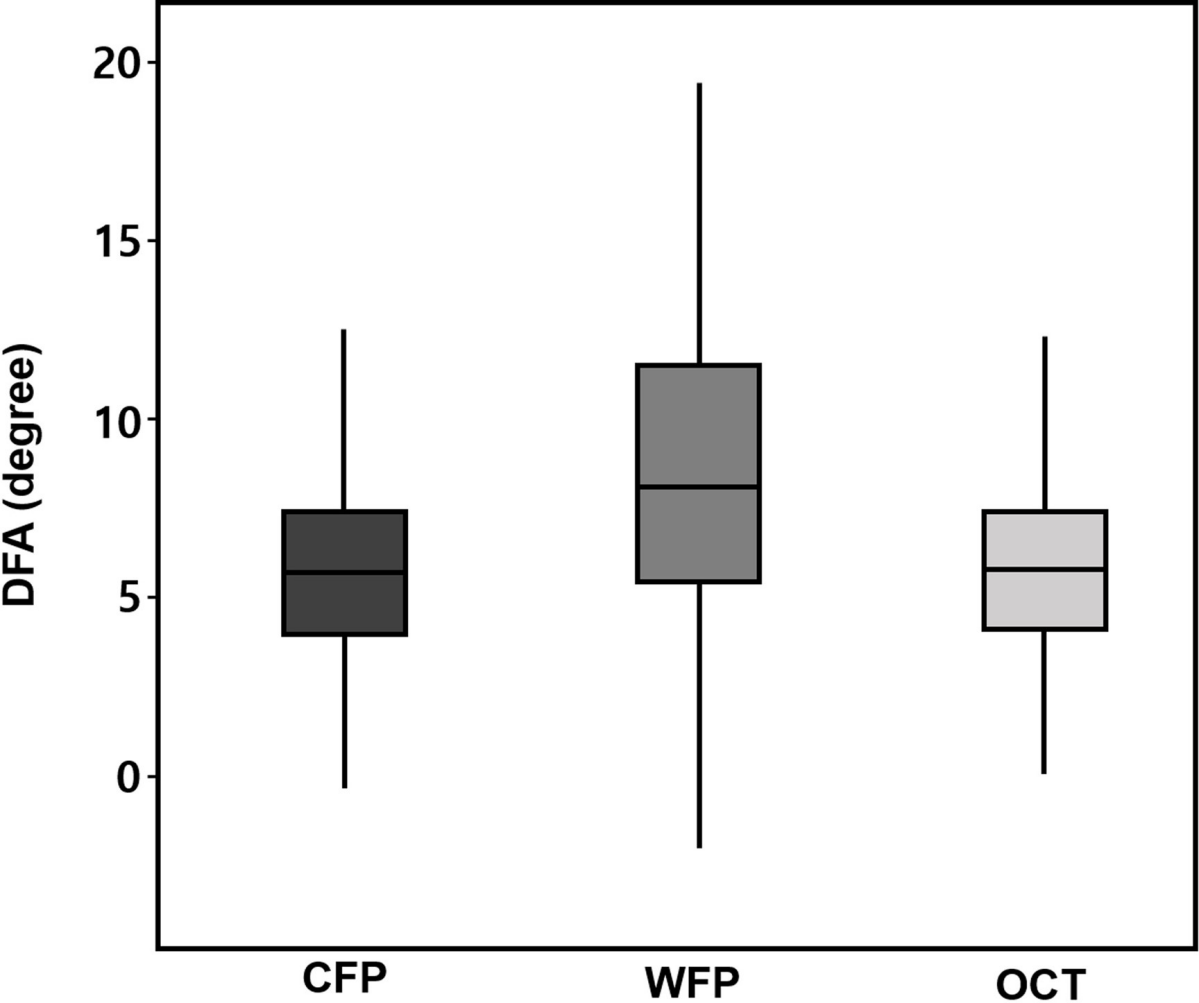
DFA values for all 278 enrolled eyes measured using Conventional Fundus Photography (CFP), Wide-field Fundus Photography (WFP), and Optical Coherence Tomography (OCT). The DFA measured using WFP was larger than when using the other two imaging modalities (*p*<0.001). The horizontal line indicates the median value, the boxes indicate the interquartile interval (25th and 75th percentiles), and the whiskers indicate the range.

**Fig 3:**
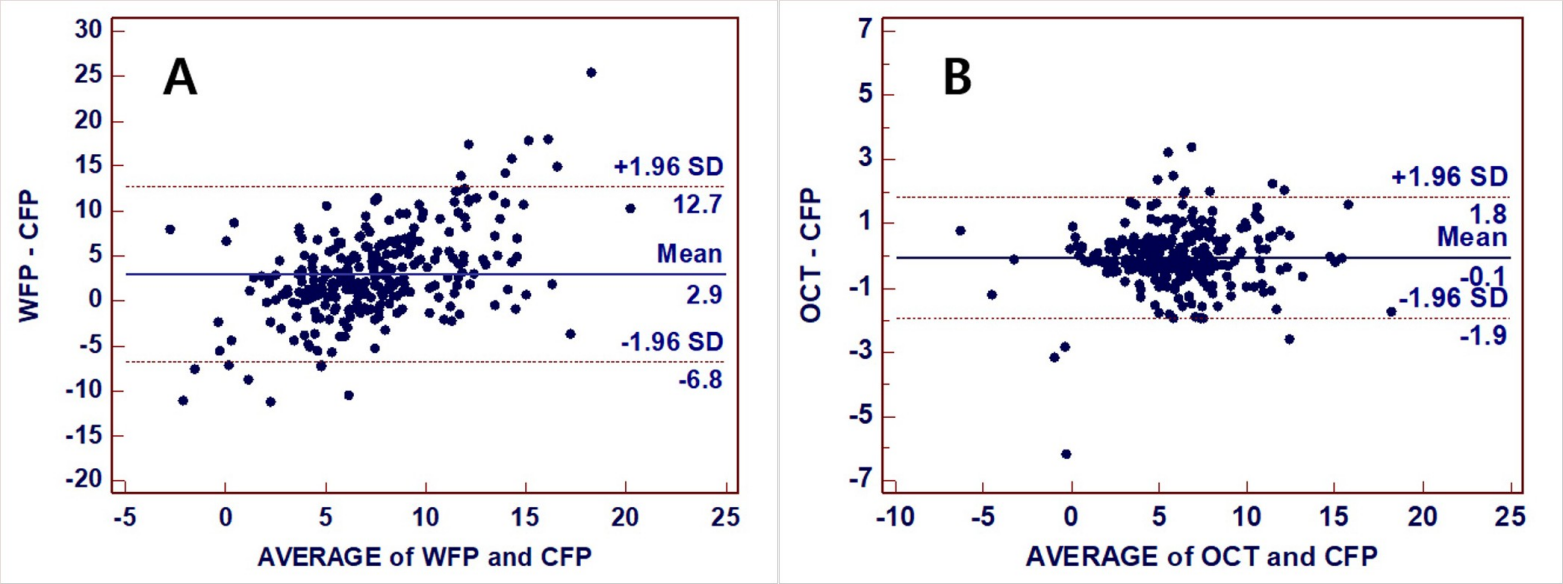
Bland-Altman plots of the agreement of the DFA between CFP and WFP and between CFP and OCT. **(A)** Differences of the DFA between WFP and CFP plotted against the mean of the two measurements. The mean difference was 2.9°, and the 95% confidence interval (CI) (1.96 × standard deviation) was from –6.8° to +12.7°. **(B)** The mean difference of the DFA between OCT and CFP plotted against the mean of the two measurements. The mean difference was –0.1° and the 95% CI was from –1.9° to +1.8°. SD, standard deviation.

When analyzing all enrolled 278 eyes according to the photographic methods, DFA was 5.98±3.22° for CFP (interquartile range [IQR] 4.03–7.59 and median 5.79), 8.90±5.40° for WFP (IQR 5.53–11.82 and median 8.31), and 5.93±3.30° for OCT (IQR 4.03–7.56 and median 5.86). The DFA measured using WFP was larger than when using the other two imaging modalities (CFP and OCT) (*p*<0.001), while it did not differ between CFP and OCT (*p* = 0.199) (Fig 2). A Bland-Altman plot of the mean paired difference was constructed to assess the agreement between WFP, OCT, and CFP (Fig 3). The 95% agreement limit for each comparison was from –6.8° to 12.7° for the comparison of WFP and CFP (Fig 3A), and from –1.9° to 1.8° for the comparison of OCT and CFP (Fig 3B). This shows that there was greater variation in the DFA measured using WFP.

## Discussion

**Fig 4:**
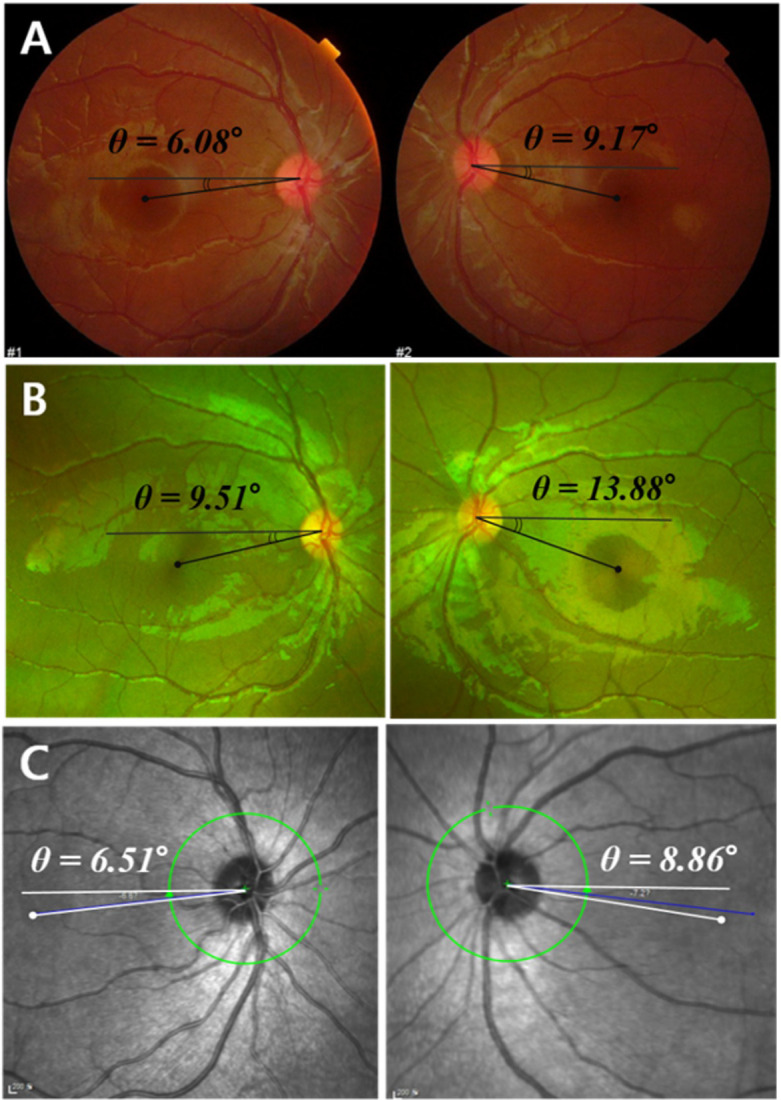
Examples of differences in the DFA according to fundus photographic methods in the same patient. The DFAs measured in the right and left eyes were 6.8° and 9.17°, respectively, for CFP **(A)**, 9.51° and 13.16° for WFP **(B)**, and 6.51° and 8.86° for OCT **(C)**. The amount of ocular extorsion was measured as being larger for WFP.

The aim of the study was to compare measurements of the cyclotorsional angle made using different photographic methods under pathological (IXT and SOP patients) and normal conditions. Our results showed that there was no significant difference in the cyclotorsional angle between CFP and OCT. However, cyclotorsional angle measured using WFP was different from angle measured using the other two imaging modalities (CFP and OCT) in all three groups. The DFA measured using WFP tends to be larger, and so this method cannot be used interchangeably with CFP and OCT. An example of one patient's images on the three imaging modalities is shown in Fig 4.

Cyclotorsion has been measured using various subjective methods, including the double Maddox rod test, Bagolini lenses, and the synoptophore [13, 14]. All of these tests require normal binocularity and the cooperation of the subject, and they are not reliable in cases of abnormal retinal correspondence or severe amblyopia. Moreover, there is a subjective sensory adaptation to fundus torsion over time, particularly if the cyclotorsion appears early during childhood [15]. Also, it is difficult to measure in young children because it requires reliable subjective responses.

Cyclotorsional analysis—especially in cases of abnormal sensory function—requires more-objective methods using anatomical ocular landmarks. The gold-standard test for the objective measurement of ocular torsion is the fundus photographic method using the DFA [10, 16]. The anatomical landmarks of the retina, fovea, and optic disc seem to be relatively constant, so the DFA measured using fundus photography is less likely to be affected by the intrarater/interrater variability, thereby producing reliable and reproducible results. Also, the DFA does not change with the duration of the follow-up [17, 18].

In the present study we applied nonmydriatic photographic methods in a binocular viewing condition. Fundus photography without pupil dilation has the advantage of preserving a more-physiological viewing condition. In an undilated exam, patients are able to maintain a clear image and get better fixation and lock on an image and keep the eyes in position. Whereas in a dilated exam, a blurred image may lead to further ocular rotation due to loss of fixation. Also, it is a noninvasive test that is easy and quick to perform, especially in children [7]. We were able to perform these tests on patients as young as 3 years. Also, pharmacological dilation tends to be inconvenient for both the medical practitioner and the patient, with the dilating drops taking about 30 minutes to take effect and the patient experiencing blurred vision and light sensitivity for up to several hours after inducing dilation. Furthermore, pharmacological dilation prevents subsequent ophthalmological and neurological examinations (e.g., of the pupils and accommodation tests) for several hours, which can be undesirable when monitoring inpatients with critical neurological diseases [19].

In our study, the physiological DFA measured using CFP in the control group was 5.55±2.92°. This result is consistent with previous reports of the DFA ranging from 5.6° to 7.0° in normal subjects, as the fovea is located within the lower one-third of the optic disc [17, 20, 21]. Also, the DFA did not vary with age in our control group. This result is also consistent with previous studies reporting that the DFA did not appear to vary with age [18, 20].

OCT can assess the presence and amount of cyclotorsion in a natural viewing condition. Its infrared imaging capability can produce images of high quality without requiring either pupil dilation or exposure to bright flashes of light. Also, a macular line scan—a horizontal line that passes through the fovea—is seen in the infrared images. The macular cross-section scan confirms the exact position of the fovea in relation to the optic disc removing any concerns of incorrect placement of line ‘b’ in the Fig 1 [8].

We found that there was no significant difference between OCT and CFP, with the DFA measured using OCT being strongly correlated with that measured using CFP in all three groups. Thus, it can be regarded that measurements of the DFA made using OCT and CFP are interchangeable (Fig 3B). In addition, the fovea-to-optic disc alignment function of OCT could be useful for automatically measuring the cyclotorsion with the aid of the protractor software provided by the device manufacturer. Lee and Kim reported that the DFA measured automatically using OCT software in the 400 eyes of 200 normal subjects did not differ from the results obtained using CFP [9].

Wide-field imaging modalities are becoming increasingly popular. In contrast to CFP providing only a 45° field of view focused on the posterior pole, WFP can image a field of view of up to 200° without requiring pupil dilation. Wide-field imaging provides valuable information about the peripheral vasculature and other retinal lesions that might otherwise be missed when using traditional imaging systems. WFP has been used successfully in pediatric ophthalmology for evaluating various retinal disorders, such as Coat’s disease and premature retinopathy [19].

A particularly interesting finding of the present study was that the DFA measured using WFP was larger than the values obtained using CFP and OCT. Fundus extorsion tends to be exaggerated in WFP (Figs 2 and 4). There are several possible explanations for this finding. First, while the optimized optical design for visualizing a wide visual field is advantageous for the periphery, it is also associated with barrel distortion problems as well as low magnification of the fovea. The barrel distortion has come from the difference of refractive index among the center and verge of the lens. This distortion can make straight lines bulge toward the edges of the image. Thus, The amount of ocular extorsion could be measured as being larger for WFP with wide-angle lens [22]. Second, visualizing the entire retina from the center to the ora serrata through a pupil of size 3–4 mm requires careful alignment of the axes of the camera lens and the eye, and the examiner has to position the eye of the patient very close to the machine. During this process the head could be slightly turned to the side in order to prevent the nose from being compressed. However, keeping the patient’s head straight is critical, since the torsion can vary with head tilting. We could anticipate that small shifts in the orientation of the macula might have exaggerated the cyclotorsion. Third, WFP has no internal fixator, while CFP and OCT do have this. Although the examiner asks the patient to look straight ahead, measurement errors are possible when the fixation or gaze direction are not strictly centered.

Additionally, in nonmydriatic fundus photography, if the accurate locations of the optic disc and macula are difficult to determine due to macular artifacts or poor photography clarity, the clinician may consider using alternative landmarks. Parsa and Kumar recently suggested that rotation of the retinal vascular arcades provides landmarks for use as an accessory sign of ocular torsion [23]. Real-time iris registration as used in refractive surgery can also be used to assess ocular torsion, but this is expensive, time-consuming, and less accurate than fundus photographic methods [24].

This study was subject to several limitations. There were only 10 patients with SOP when comparing to 44 patients with IXT and 85 normal control subjects. In addition, the measurements were made in this study under the condition of binocular viewing. Although objective measurements of fundus photography are less likely to be affected by the viewing conditions, the effect of pupil dilation and monocular viewing conditions compared to undilated binocular viewing conditions on ocular torsion still need to be determined.

In conclusion, fundus analysis based on the DFA is a reliable tool for objectively assessing ocular torsion. However, measured DFA values could differ with the photographic method even in the same patient. We confirmed that measuring ocular torsion using OCT showed a strong correlation with the results obtained using previous methods with CFA. We found, however, that there was only a weak correlation between WFP and the other two methods (CFP and OCT). Since the DFA measured using WFP tends to be larger than when using the other two methods, clinicians should take special care to not overestimate ocular extorsion when interpreting images obtained using WFP. Further studies are needed to identify standardized head-positioning and image-adjusting techniques that could minimize the variations in cyclotorsion measurements.

## Supporting information

S1 File (XLSX)
